# Supplementary material for: Characterization of the Lower Airways and Oral Microbiota in Healthy Young Persons in the Community
Source: Biomedicines. 2023 Mar 10;11(3):841. doi: 10.3390/biomedicines11030841 (PMC10045431; doi:10.3390/biomedicines11030841)
Supplement: Supplementary file 1 [file biomedicines-11-00841-s001.zip › biomedicines-2199948-supplementary.pdf]

# Characterization of the Lower Airways and Oral Microbiota in Healthy Young Persons in the Community

**Fernando Sergio Leitao Filho [1,2], Carli Monica Peters [3], Andrew William Sheel [3], Julia Yang [1,2], Corey Nislow [4], Stephen Lam [1,2,5], Janice M. Leung [1,2] and Don D. Sin [1,2,*]**

[1] UBC Centre for Heart Lung Innovation, St. Paul's Hospital, 1081 Burrard Street, Room 166, Vancouver, BC V6Z 1Y6, Canada; fernando.studart@hli.ubc.ca (F.S.L.F.); julia.yang@hli.ubc.ca (J.Y.); slam2@bccancer.bc.ca (S.L.); janice.leung@hli.ubc.ca (J.M.L.)
[2] Division of Respiratory Medicine, Department of Medicine, University of British Columbia, Vancouver, BC V1V 1V7, Canada
[3] School of Kinesiology, Faculty of Education, University of British Columbia, Vancouver, BC V5Z 1M9, Canada; carli.peters@hli.ubc.ca (C.M.P.); bill.sheel@ubc.ca (A.W.S.)
[4] Faculty of Pharmaceutical Sciences, University of British Columbia, Vancouver, BC V1V 1V7, Canada; corey.nislow@ubc.ca
[5] British Columbia Cancer Agency, Vancouver, BC V5Z 4E6, Canada
* Correspondence: don.sin@hli.ubc.ca; Tel.: +1-(604)-806-8395; Fax: +1-(604)-806-8722

## DNA extraction and PCR amplification

DNA was extracted from 500 µL sample aliquots using the DNeasy Blood & Tissue Kit (QIAGEN®, Toronto, ON, Canada). Each aliquot was centrifuged for 10 minutes at 5000 × g to form a pellet. After the supernatant was discarded, the pellet was resuspended in 180 µL of Buffer ATL and DNA was extracted according to the manufacturer's instructions· The concentration of DNA was measured using the Quant-iT® High-Sensitivity dsDNA Assay Kit (Life Technologies, Carlsbad, CA, USA).

Total 16S rRNA libraries were quantified using a droplet digital PCR assay (Bio-Rad Laboratories®, Mississauga, Canada). PCR amplification was performed using the AccuPrime® Taq DNA Polymerase System (Life Technologies®, Carlsbad, CA, USA). Primers targeting the variable V4 region of the 16S rRNA gene were used.

Each 20 µL PCR reaction contained 20 pmol of each indexed primer, 2 µL of 10× PCR Buffer II, 0.15 µL of Taq DNA Polymerase, and 20 ng of DNA template. PCR reactions were placed in a T100 Thermal Cycler (Bio-Rad Laboratories®, Mississauga, ON, Canada). The touchdown PCR program was as follows: 95°C for 2 min; 20 cycles of 95°C for 20 s, 60°C for 15 s (decreasing by 0.3°C per cycle), and 72°C for 90 s; 20 cycles of 95°C for 20 s, 55°C for 15 s, and 72°C for 90 s; and, lastly, 72°C for 5 min. The size of the obtained amplicons was checked by gel electrophoresis with a 1.5% agarose gel containing SYBR Safe DNA Gel Stain (Life Technologies®, Carlsbad, CA, USA). The presence of a band on the agarose gel indicated successful amplification. Before pooling the PCR amplicons, cleanup and normalization were performed using the SequalPrep® Normalization Plate Kit (Applied Biosystems®, Frederick, MD, USA). The pool of PCR reaction products was checked for size and quality using a DNA 1000 Kit with the 2100 Bioanalyzer system (Agilent Technologies®, Santa Clara, CA, USA) and diluted to 4 nM with 10 mM Tris-Cl pH 8.5 before sequencing.

## Sequencing accuracy

A mock community (HM-782D) containing 20 bacterial species was provided to evaluate the sequence quality (BEI Resources®, Virginia, USA). According to the *qiime quality-control evaluate-seqs* plugin function [1], we detected only 5 mismatches and no gaps (identified in only 2/20 ASVs from our MOCK community), resulting in a sequencing accuracy of ~99.5%.

**Table S1:** Taxonomic annotations at the phylum and genus levels for amplicon sequence variants (ASVs) identified as potential contaminants according to the Decontam R package.

| ASV | Phylum | Genus | Species |
|---|---|---|---|
| ASV1 | *Proteobacteria* | *Escherichia-Shigella* | NA |
| ASV2 | *Cyanobacteria* | *Chloroplast* | NA |
| ASV3 | *Cyanobacteria* | *Chloroplast* | NA |
| ASV4 | *Proteobacteria* | *Sphingomonas* | NA |
| ASV5 | *Proteobacteria* | NA | NA |
| ASV6 | *Proteobacteria* | *Tepidiphilus* | *uncultured_bacterium* |
| ASV7 | *Firmicutes* | *Mogibacterium* | *uncultured_rumen* |
| ASV8 | *Actinobacteriota* | *Corynebacterium* | NA |
| ASV9 | *Verrucomicrobiota* | *Chthoniobacter* | NA |
| ASV10 | *Patescibacteria* | *Candidatus Lloydbacteria* | *Candidatus Lloydbacteria* |
| ASV11 | *Firmicutes* | *Mogibacterium* | *uncultured rumen* |
| ASV12 | *Cyanobacteria* | *Chloroplast* | NA |
| ASV13 | *Proteobacteria* | *Brevundimonas* | NA |
| ASV14 | *Proteobacteria* | *Bosea* | NA |
| ASV15 | *Proteobacteria* | *Legionella* | NA |
| ASV16 | *Proteobacteria* | *Neisseria* | NA |
| ASV17 | *Verrucomicrobiota* | *Candidatus Xiphinematobacter* | *uncultured Candidatus* |
| ASV18 | *Bdellovibrionota* | *Oligoflexus* | *metagenome* |
| ASV19 | *Proteobacteria* | *Methylobacterium-Methylorubrum* | NA |
| ASV20 | *Proteobacteria* | *Pedomicrobium* | |
| ASV21 | *Patescibacteria* | *Candidatus Nomurabacteria* | *uncultured bacterium* |
| ASV22 | *Patescibacteria* | *Candidatus Azambacteria* | *Parcubacteria bacterium* |
| ASV23 | *Bacteroidota* | *Porphyromonas* | *uncultured bacterium* |
| ASV24 | *Campylobacterota* | *Campylobacter* | NA |
| ASV25 | *Firmicutes* | *Butyrivibrio* | *Eubacterium* sp. |
| ASV26 | *Bacteroidota* | *Prevotella-7* | *Prevotella enoeca* |
| ASV27 | *Cyanobacteria* | *Chloroplast* | NA |
| ASV28 | *Firmicutes* | *Parvimonas* | *uncultured bacterium* |
| ASV29 | *Bacteroidota* | *Tannerella* | *uncultured bacterium* |
| ASV30 | *Desulfobacterota* | *Desulfobulbus* | NA |

*Includes both control and clinical samples, as follows: 1) clinical samples: bronchial brushings, n=25; bronchoalveolar lavage (BAL) samples, n=25; oral wash samples, n=25; 2) control samples: extraction negatives, n=5; bronchoscope channel washes, n=23; CytoLyt controls, n=25. The "frequency" method in the Decontam R package [2] was used to identify possible contaminants; in this method, the frequency distribution of each sequencing feature as a function of the input DNA concentration is used to identify contaminants. Legend: NA = not applicable.

**Figure S1:** Comparison of microbial structures (beta-diversity) between clinical samples (BB, BAL, and OW) and control samples (EN, NTC, BCW, and CC).

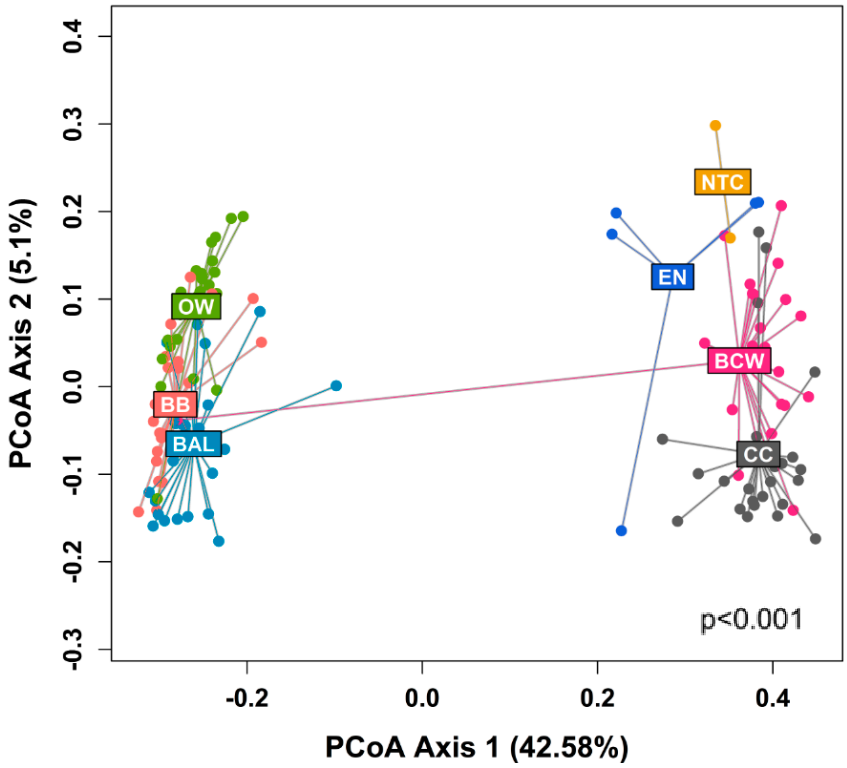


Permutational Multivariate Analysis of Variance (PERMANOVA; function adonis in the vegan R package [3]) based on Generalized Unifrac distance (alpha=0.5) [4] was used for comparison of microbial structures among specimen types. Legend: PCoA = Principal Coordinates Analysis; BB = bronchial brushing (n=25); BAL = bronchoalveolar lavage (n=25); OW = oral wash (n=25); BCW = bronchoscope channel wash (n=23); EN = extraction negative (n=5); NTC = non-template control (n=2); CC = CytoLyt controls (n=25). The BCW controls were retrieved by flushing 40 mL of sterile 0,9% saline through the bronchoscope before the procedure into a sterile specimen cup. The EN controls contained only DNA extraction reagents, whereas NTC consisted of ultra-purified water instead of a DNA sample during the PCR reaction, and the CC controls contained only CytoLyt® solution. Pairwise PERMANOVA results are displayed in Table S2.

**Table S2:** Pairwise PERMANOVA results based on a Generalized Unifrac distance considering clinical samples (n=75) and control samples (n=55).

| **Group 1** | **Group 2** | **Sample size** | **Permutations** | ***p-value*** | ***adj. p-value*** |
|---|---|---|---|---|---|
| BAL | BB | 50 | 999 | 0.009 | 0.01 |
| BAL | BCW | 48 | 999 | 0.001 | 0.002 |
| BAL | CC | 50 | 999 | 0.001 | 0.002 |
| BAL | EN | 30 | 999 | 0.001 | 0.002 |
| BAL | OW | 50 | 999 | 0.001 | 0.002 |
| BAL | NTC | 27 | 999 | 0.003 | 0.005 |
| BB | BCW | 48 | 999 | 0.001 | 0.002 |
| BB | CC | 50 | 999 | 0.001 | 0.002 |
| BB | EN | 30 | 999 | 0.001 | 0.002 |
| BB | OW | 50 | 999 | 0.001 | 0.002 |
| BB | NTC | 27 | 999 | 0.002 | 0.003 |
| BCW | CC | 48 | 999 | 0.001 | 0.002 |
| BCW | EN | 28 | 999 | 0.013 | 0.013 |
| BCW | OW | 48 | 999 | 0.001 | 0.002 |
| BCW | NTC | 25 | 999 | 0.006 | 0.007 |
| CC | EN | 30 | 999 | 0.005 | 0.007 |
| CC | OW | 50 | 999 | 0.001 | 0.002 |
| CC | NTC | 27 | 999 | 0.008 | 0.009 |
| EN | OW | 30 | 999 | 0.001 | 0.002 |
| EN | NTC | 7 | 999 | 0.493 | 0.49 |
| OW | NTC | 27 | 999 | 0.004 | 0.006 |

Legend: BB = bronchial brushing (n=25); BAL = bronchoalveolar lavage (n=25); OW = oral wash (n=25); BCW = bronchoscope channel wash (n=23); EN = extraction negative (n=5); NTC = non-template control (n=2); CC = CytoLyt control (n=25). p-values were based on the Permutational analysis of variance (PERMANOVA) method [5], and pairwise PERMANOVA results were obtained according to the Benjamini-Hochberg method [6]. The PERMANOVA results were obtained directly from QIIME 2™ (based on the qiime diversity plugin) .

**Table S3:** Most abundant bacterial species identified in each microbiome compartment.

| | Bronchial Brushings | | | BAL samples | | | Oral Wash samples | | |
|---|---|---|---|---|---|---|---|---|---|
| **Species** | **Mean** | **Median** | **Q1; Q3** | **Mean** | **Median** | **Q1; Q3** | **Mean** | **Median** | **Q1; Q3** |
| *Veillonela dispar** | 14.0 | 14.7 | 9.2; 7.9 | 16.1 | 15.5 | 13.9; 18.1 | 15.5 | 15.0 | 11.4; 16.8 |
| *Haemophilus parainfluenzae** | 4.2 | 3.1 | 1.7; 6.2 | 6.3 | 5.5 | 4.5; 7.2 | 15.3 | 16.3 | 11.7; 18.7 |
| *Prevotella jejuni** | 12.0 | 9.6 | 5.9; 17.5 | 9.2 | 8.8 | 6.9; 11.9 | 6.9 | 5.4 | 3.8; 18.7 |
| *Prevotella melaninogenica* | 7.4 | 7.0 | 2.6; 10.9 | 7.8 | 7.0 | 4.1; 9.1 | 11.1 | 10.9 | 6.7; 12.5 |
| *Prevotella pallens* | 8.4 | 6.1 | 3.1; 9.7 | 6.7 | 5.8 | 3.9; 9.3 | 5.4 | 4.0 | 3.1; 7.9 |
| *Megasphaera micronuciformis* | 3.0 | 2.7 | 0.3; 4.2 | 2.3 | 2.4 | 1.5; 3.2 | 0.6 | 0.5 | 0.2; 0.8 |
| *Prevotella salivae* | 2.5 | 2.1 | 1.0; 3.4 | 1.4 | 1.1 | 0.8; 2.1 | 1.5 | 1.1 | 0.5; 2.2 |
| *Streptococcus salivarius* | 0.5 | 0.3 | 0.1; 0.7 | 0.4 | 0.3 | 0.1; 0.5 | 0.3 | 0.1 | 0.1; 0.4 |
| *Mogibacterium pumilum* | 0.2 | 0.1 | 0.0; 0.2 | 0.3 | 0.2 | 0.1; 0.5 | 0.0 | 0.0 | 0.0; 0.0 |
| *Prevotella intermedia* | 0.9 | 0.1 | 0.0; 0.6 | 1.8 | 0.2 | 0.0; 1.1 | 0.8 | 0.1 | 0.0; 0.5 |
| *Prevotella nigrescens* | 1.2 | 0.1 | 0.0; 1.2 | 0.8 | 0.4 | 0.1; 0.9 | 0.8 | 0.3 | 0.2; 0.9 |

*Species identification only possible after performing sequence comparisons using the National Center for Biotechnology Information Basic Local Alignment Tool (NCBI BLAST, Maryland, USA) .

Definition of abbreviations: bronchoalveolar lavage; Q1: first quartile; Q3: third quartile.

**Table S4:** Relative abundance and frequency data of pathogenic genera in each microbiome compartment based on oral and lower airways samples collected from healthy subjects (n=25).

| **Taxon** | **MICROBIOME COMPARTMENT** | | | | | | **Statistics** |
|---|---|---|---|---|---|---|---|
| | **Bronchial brushings** | | **BAL samples** | | **Oral wash samples** | | |
| **Genus** | **Median** | **Observed in** | **Median** | **Observed in** | **Median** | **Observed in** | ***p-value**** |
| *Moraxella* | 0.0% | 2/25 (8.0%) | 0.0% | 1/25 (4.0%) | 0.0% | 1/25 (4.0%) | <0.001 |
| *Pseudomonas* | 0.0% | 4/25 (16.0%) | 0.0% | 2/25 (8.0%) | 0.0% | 0/25 (0.0%) | <0.001 |
| *Acinetobacter* | 0.0% | 5/25 (20.0%) | 0.0% | 0/25 (0.0%) | 0.0% | 0/25 (0.0%) | <0.001 |
| *Staphylococcus* | 0.0% | 5/25 (20.0%) | 0.0% | 0/25 (0.0%) | 0.0% | 0/25 (0.0%) | <0.001 |

Definition of abbreviations: BAL = bronchoalveolar lavage.
*P-values were calculated using a Fisher's exact test.

**Table S5:** Baseline characteristics of participants recruited into the British Columbia Cancer Agency (BCCA) cohort (n=47) [7].

| Variables | Results |
|---|---|
| Age, years | 62.9 ± 7.8 |
| Sex, female | 22 (46.8) |
| Smoking status | |
| Current smokers | 20 (42.5) |
| Former smokers | 24 (51.1) |
| Never-smokers | 3 (6.4)* |
| Pack-years | 47.0 ± 13.9 |
| COPD status | |
| COPD | 24 (51.1) |
| Non-COPD | 23 (48.9) |

Values are expressed as mean ± SD or n (%). Definition of abbreviations: COPD = Chronic Obstructive Pulmonary Disease.
*Out of the three never-smokers, two were non-COPD.

## References


1. Bolyen E, Rideout JR, Dillon MR, Bokulich NA, Abnet CC, Al-Ghalith GA, et al. Reproducible, interactive, scalable and extensible microbiome data science using QIIME 2. Nat Biotechnol. 2019;37(8):852-7.
2. Davis NM, Proctor DM, Holmes SP, Relman DA, Callahan BJ. Simple statistical identification and removal of contaminant sequences in marker-gene and metagenomics data. Microbiome. 2018;6(1):226.
3. Dixon P. VEGAN, a package of R functions for community ecology. Journal of Vegetation Science. 2003;14(6):927-30.
4. Chen J, Bittinger K, Charlson ES, Hoffmann C, Lewis J, Wu GD, et al. Associating microbiome composition with environmental covariates using generalized UniFrac distances. Bioinformatics. 2012;28(16):2106-13.
5. Anderson MJ. A new method for non-parametric multivariate analysis of variance. Austral Ecology. 2001;26(1):32-46.
6. Benjamini Y, Hochberg Y. Controlling the False Discovery Rate: A Practical and Powerful Approach to Multiple Testing. Journal of the Royal Statistical Society: Series B (Methodological). 1995;57(1):289-300.
7. Marshall EA, Filho FSL, Sin DD, Lam S, Leung JM, Lam WL. Distinct bronchial microbiome precedes clinical diagnosis of lung cancer. Mol Cancer. 2022;21(1):68.
